# Optimizing Older Adult Mental Health in Support of Healthy Ageing: A Pluralistic Framework to Inform Transformative Change across Community and Healthcare Domains

**DOI:** 10.3390/ijerph21060664

**Published:** 2024-05-23

**Authors:** Salinda Horgan, Jeanette Prorok, Katie Ellis, Laura Mullaly, Keri-Leigh Cassidy, Dallas Seitz, Claire Checkland

**Affiliations:** 1 Departments of Rehabilitation Therapy & Psychiatry, Queen’s University, Kingston, ON K7L 3N6, Canada; 2 School of Rehabilitation Therapy, Queen’s University, Kingston, ON K7L 3N6, Canada; prorkj@queensu.ca; 3 Mental Health Commission of Canada, Ottawa, ON K1R 1A4, Canada; kellis@mentalhealthcommission.ca (K.E.); lmullaly@mentalhealthcommission.ca (L.M.); 4 Department of Psychiatry, Dalhousie University, Dalhousie, NS B3H 2E2, Canada; keri-leigh.cassidy@nshealth.ca; 5 Departments of Psychiatry & Community Health Sciences, University of Calgary, Calgary, AB T2N 1N4, Canada; dallas.seitz@ucalgary.ca; 6 Canadian Coalition for Seniors’ Mental Health, Markham, ON L3R 9X9, Canada; ccheckland@ccsmh.ca

**Keywords:** older adults, mental health, conceptual framework, health prevention, health promotion, policy and planning, ecological

## Abstract

This paper describes a pluralistic framework to inform transformative change across community and healthcare domains to optimize the mental health of older adults in support of healthy ageing. An extensive review and analysis of the literature informed the creation of a framework that contextualizes the priority areas of the WHO Decade of Health Ageing (ageism, age-friendly environments, long-term care, and integrated care) with respect to older adult mental health. The framework additionally identifies barriers, facilitators, and strategies for action at macro (social/system), meso (services/supports), and micro (older adults) levels of influence. This conceptual (analytical) framework is intended as a tool to inform planning and decision-making across policy, practice, education and training, research, and knowledge mobilization arenas. The framework described in this paper can be used by countries around the globe to build evidence, set priorities, and scale up promising practices (both nationally and sub-nationally) to optimize the mental health and healthy ageing trajectories of older adults as a population.

## 1. Introduction

In the context of global population ageing, the World Health Organization (WHO) has brought international attention to the health and wellbeing of older adults [1]. Globally, older adults experience inequities in health and social circumstances that jeopardize their physical, mental, social, environmental, and spiritual health [2] and truncate healthy ageing trajectories [3]. To promote conditions that enable and sustain healthy ageing and promote equitable health and care outcomes, the WHO launched the Decade of Healthy Ageing campaign [4], the goal of which is to improve the functional ability of all older adults such that they can age healthily. To achieve healthy ageing, older adults require the functional ability to experience health (i.e., absence of illness or infirmity) and wellbeing (i.e., state or quality of being and feeling healthy) in their day-to-day lives [4]. Functional ability (i.e., a combination of a person’s intrinsic capacity, their environment, and interpersonal interactions) is a critical attribute that impacts their ability to meet basic needs; learn; make decisions; build and maintain relationships; and contribute socially [3]. 

Mental health can either enable (or disable) functional ability [5]. Mental health, as it underpins overall functioning, is a critical stimulus that shapes the overall ability to age well [6,7]. Mental health falls along a continuum that extends *across* mental *wellness* (i.e., presence of positive mental wellbeing), mental health *concerns* (i.e., diminished cognitive, emotional capacity that interferes with enjoyment of life), and mental *illness* (i.e., mental health disorders that interfere with day-to-day functioning) and is *interconnected* with physical, emotional, cognitive, social, and environmental health [6]. This definition promotes a view of mental health (similar to that of physical health) that recognizes and considers its’ complex and dynamic nature across situations and circumstances [5]. It supports the notion that *all* people experience mental health and, depending on physiological and life scenarios, these experiences may fluctuate across the continuum from wellness to illness, and back. Mental health is further conceptualized as an interconnecting operant that axiomatically shapes overall health. Good mental health is thus a necessary ingredient for achieving healthy ageing. 

Unfortunately, when it comes to older adults, mental health promotion and mental illness prevention and treatment are often overlooked [8]. This results in a lack of services and support across community and healthcare settings that consider and respond to their mental health needs [9]. To some degree, this is due to complex health scenarios that complicate the prevention and management of mental health concerns and illnesses in this population [8]. However, it is also due to the multitudinous layers of stigma and discrimination in community and healthcare environments that de-prioritize the values, goals, and capabilities (i.e., requirements) of older adults [10,11]. 

Fortunately, there is substantial evidence that strategic efforts to construct environments to better meet the requirements of older adults can reduce and potentially eradicate the inequities that exacerbate mental health harms and impede healthy ageing [9,10]. To optimize mental health as a foundation of healthy ageing, transformative change is required across community and healthcare domains [11,12]. It is only through strategic efforts to plan, incentivize, and action transformations in and across traditionally siloed community and healthcare domains that mental health harms can be ameliorated [11,12]. 

Currently, there is substantial evidence that older adults have insufficient access to community and healthcare environments that promote their mental health [2,13]. The social and systemic disadvantages that shape their lived reality of ageing, foster mental health, quality of life, and healthy ageing inequities [8]. This places older adults at heightened risk of [2] depression [14], anxiety [15], addictions [14,15], social isolation, and loneliness [16]. The outcomes associated with mental health and care inequities pose detrimental costs to older adults, families, communities, and the health system [17,18]. In contrast, when older adults have access to environments that promote their mental health, outcomes associated with healthy ageing are shown to improve [5].

In this context, of growing challenges and opportunities for healthy ageing, the Decade of Healthy Ageing Baseline Report [1] (i.e., the Decade) can provide a springboard to envision strategic efforts to plan, incentivize, and action transformations in and across community and healthcare domains to optimize the mental health of older adults. The Decade identifies four priority areas (across community and healthcare domains) in need of immediate action to help older adults age healthily: ageism, age-friendly communities, integrated care, and long-term care. This uniquely pluralistic lens is optimal for conceptualizing a whole-system and population health approach to older adult mental health. 

Additional efforts are needed to build on and extend this work with respect to mental heath. Further efforts are needed to (a) situate and contextualize the priority areas of the Decade with specific relevance to the mental health of older adults, (b) identify barriers, facilitators to inform change efforts across policy, practice, education and training, research, and knowledge mobilization arenas, and (c) determine strategies for action to operationalize change at macro (social/system), meso (services/supports), and micro (older adults) levels of influence.

## 2. Current Study

The aim of the current study was to develop a conceptual (analytical) framework that builds on the areas of the *Decade* to inform transformative change efforts across community and healthcare domains to optimize the mental health of older adults in support of healthy ageing. Conceptual frameworks provide a structured and comprehensive view of a topic or phenomenon while highlighting nuanced relationships, connections and interconnections. The framework described in this paper and is referred to hereon as the Framework. An extensive review and analysis of the literature informed the construction of a pluralistic (i.e., applicable in and across community/healthcare domains) Framework that contextualizes the areas of the *Decade* with respect to older adult mental health. The framework additionally identifies barriers, facilitators, and strategies for action at macro (social/system), meso (services/supports), and micro (older adults) levels of influence. The Framework is intended as a tool to guide transformative policy, practice, education and training, research, and knowledge mobilization efforts (at national and sub-national levels) to optimize the mental health and healthy ageing trajectories of older adults as a population. 

## 3. Method

The conceptual Framework was developed through a systematic process that included the following steps: (1) an extensive narrative review of the literature pertaining to older adult mental health in each of the four priority areas of the Decade; (2) an interpretive thematic analysis to contextualize each area with respect to the mental health of older adults; (3) a content analysis to identify barriers, facilitators and strategies at macro (social/system), meso (services/supports) and micro (older adults) levels of influence.

*STEP 1*: An extensive narrative review of the literature was conducted to obtain a broad overview of the current knowledge regarding the mental health, care needs, and related health inequities of older adults in and across the four action areas of the Decade (ageism, age-friendly environments, long-term care, and integrated care). The review was informed by the approach described by Arksey and O’Malley [19] (2005) and adapted by Tricco et al. [20] (2018). The process consisted of the following steps: (1) identifying the research question; (2) searching for relevant studies; (3) selecting studies; (4) charting the data; (5) collating, summarizing, and reporting the results; (6) consulting with key stakeholders and translating knowledge. The research question guiding the review was, “*What are the specific mental health and care needs and related health and social inequities of older adults in and across the four action areas of the Decade?*” The search strategy was developed in consultation with a University Health Sciences Librarian. Search terms included variations and truncations of the following concepts: older adults, mental health or illness, wellness, stigma, ageism, LTC, integrated care, age-friendly environments, health inequality, mental health needs, and social determinants of health. The following databases were searched: MEDLINE (Ovid), EMBASE, PsycINFO, Academic Search Complete, and Google Scholar. A Google search and citation tracking were conducted to identify relevant government/organizational (grey literature) documents. Members of a project advisory committee were consulted to assist in identifying relevant literature. To be included in the review, documents had to meet the following criteria: (i) written in either English or French; (ii) published between 2017 and 2022; (iii) peer-reviewed or grey literature (articles in peer-reviewed journals or government/organizational documents); (iv) explicitly describe relevant the mental health needs of older adults or related health inequities; and (v) be applicable to one or more of the four action areas of the Decade. 

*STEP 2:* An iterative, thematic analysis was employed to synthesize the narrative thread of the extracted data in and across the four action areas. Thematic analysis is a qualitative technique that can be used to interpret patterns and associated meanings that arise in the existing literature to answer the research question. 

*STEP 3*: A content analysis was employed to identify specific facilitators, barriers, and strategies for action. Content analysis is a qualitative technique that can be used to systematically code the data from literature reviews to classify key categories of interest. 

## 4. Results

*SEARCH RESULTS:* The initial search produced 1632 hits. A total of 1176 documents were initially removed based on duplication and title/abstract relevance. A full-text review was conducted on the remaining 456 documents. A total of 164 documents met the inclusion criteria. 

*FRAMEWORK:* The findings of the literature review informed the development of a *Pluralistic Framework to Inform Transformative Change Across Community and Healthcare Domains to Optimize the Mental Health of Older Adults in Support of Healthy Ageing*. 

### 4.1. Overview and Structure

**Figure 1:**
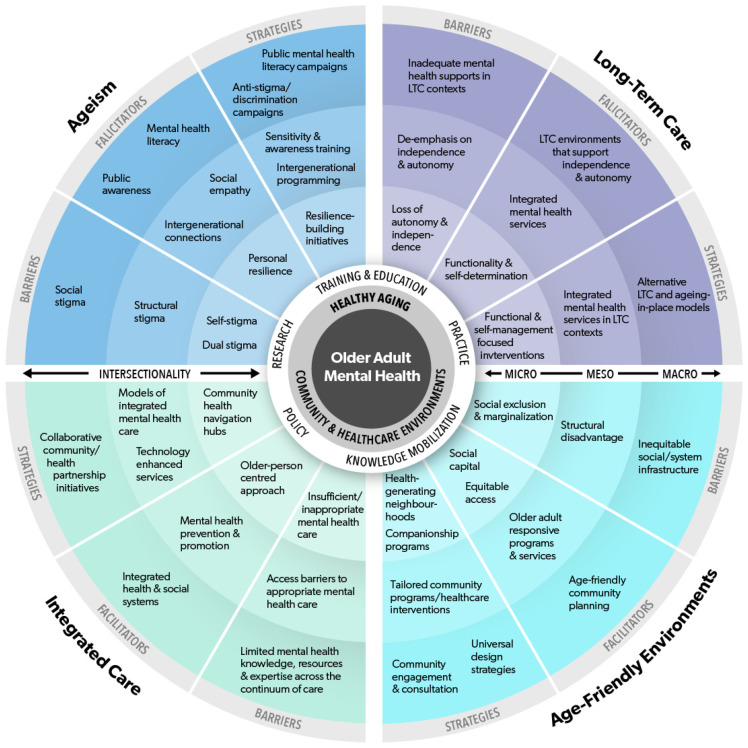
A pluralistic Framework to inform transformative change across community and healthcare domains to optimize the mental health of older adults in support of healthy ageing.

This section of the paper describes a pluralistic Framework to inform transformative change across community and healthcare domains to optimize the mental health of older adults in support of healthy ageing (referred to as the Framework). Figure 1 provides a visual depiction of the Framework. The following description of the Framework is aligned with the visual and begins at the core (older adults) and moves outward to describe each domain in greater detail. Situated at the *core of the Framework* is the population of older adults, inclusive of their mental health experiences (which can fall anywhere along the continuum of mental wellness–mental health concerns–mental illnesses). *Encircling the core are healthy ageing experiences* in and across community and healthcare environments that are supported (or impeded) by overall mental health. The intertwined mental health and healthy ageing experiences of older adults are in turn encircled by the *four areas of the Decade* (i.e., community and healthcare domains). Each area (or domain) is *contextualized in terms of three components* (i) mental health harms (barriers), (ii) optimizing mental health (facilitators), and (iii) strategies for action. The different components are then described at macro (social/system), meso (services/supports), and micro (older adults) *levels of influence*. The macro level encompasses broader social and healthcare systems. The meso level comprises community programs and healthcare services and the micro level pertains to older adults and caregivers. Barriers, facilitators, and strategies are described in order from macro to micro (rather than micro to macro) because the macro level represents the umbrella infrastructure that shapes meso-level conditions and micro-level experiences. 

### 4.2. Core: Mental Health and Healthy Ageing Experiences of Older Adults

Older adults are situated at the core of the Framework. It is recognized that *all older adults* live in various states of mental health that are either optimal (mental wellness) or recoverable (mental health concerns and illnesses) in the sense that people can be moved along the continuum (via informal and formal therapeutic interventions) to bring them closer to an optimal state of mental health. It is further noted that ageing experiences (i.e., how older adults perceive the overall quality of their everyday lives) both impact and are impacted by one’s mental health. Thus, healthy ageing experiences bolster mental health, and positive mental health bolsters healthy ageing. It is possible for all older adults to experience improvements in their overall mental health. Moreover, older adults can achieve degrees of mental wellness even while dealing with mental health concerns and/or illnesses. This requires a holistic approach to services and support that incorporates interconnected mental health promotion/prevention, management, and treatment strategies that enable older adults to function well and enjoy a good quality of life. 

### 4.3. Domain 1: Ageism 

Ageism falls under the broader umbrella of stigma. Ageism refers to socially constructed and discriminatory ways of thinking about and acting toward older adults that foster inequitable social, economic, political, educational, and psychological outcomes. Older adults face ageist, health-related, and other stigmas that ultimately devalue and position them outside of mainstream society [4]. 

#### 4.3.1. Ageism and Mental Health

Age-related physiological and neurological declines mean that older adults are increasingly seen (and treated) as ‘other’. Studies show that ageist stigmas have a negative impact on the mental health of the older adult population. Ageist attitudes and mental health stigmas are commonly intertwined. This is seen in the frequent (and mistaken) assumption that non-normative behaviors in older adults (linked to hearing difficulty, visual impairments, slowed cognitive processing) are symptoms of mental health concerns and illnesses (e.g., signs of depression, anxiety, or dementia). Alternatively, ageism promotes the (mistaken) belief that mental health concerns and/or illnesses (i.e., mental health issues) are a natural part of the ageing process [21], leading to disparities in the prevention and treatment of mental health issues in this population [22,23]. By contrast, having a more positive outlook on ageing has been linked with increased longevity at both individual and community levels [5].

#### 4.3.2. Mental Health Harms

Macro (community and healthcare systems): *Social stigma* consists of publicly displayed negative attitudes, stereotypes, and behaviors. Many harmful ideas associated with ageing (e.g., as people age, they are less capable, less interested in physical activity and social participation, and have less to offer society) are reflected in the attitudes and behaviors exhibited towards older adults [24]. The extended repercussions (e.g., exclusion, marginalization, isolation) increase the probability that older adults will be less motivated to participate socially [25,26], suffer psychological distress [27,28], and experience social isolation and loneliness [27]. Mental health and ageist stigmas have also been shown to have a direct impact on cognitive performance [29], addictions [30], and suicidality [25] in older adults. 

Meso (community programs and healthcare services): Social stigma can lead to inequitable consideration of the mental health of older adults when planning and implementing programs and services [31,32]. *Structural stigma* cultivates unequal conditions and circumstances in and across social, economic, educational, and health domains on the basis of age and age-related health issues [33,34]. The consequent systemic disadvantages can be seen in scenarios of forced retirement, unaffordable housing options, inaccessible public transportation [35], and untimely mental health care [36]. These disadvantages perpetuate mental health harms in the form of psychological distress [27,28], chronic illness [37], and premature mortality [25,38]. 

Micro (older adults): Older adults are vulnerable to the harmful effects of *self-stigma* [27]. Stigma-induced social interactions [29,32] inhibit the quality and quantity of contact with others [39] and diminish opportunities for meaningful social interactions [40]. There is a direct association between internalized ageist and mental health stigmas, and feelings of low self-worth [32], reduced levels of social participation [34], and ineffective coping [41]. *Dual stigma* occurs when a person is continuously exposed to multiple sources of stigma [42]. Older adults who experience mental health issues are exposed to stigma that is attributable to both their age and health [43]. The number of stigmas a person endures can have a cumulative effect on overall emotional wellbeing [44,45]. 

#### 4.3.3. Optimizing Mental Health Outcomes 

Macro (community and healthcare systems): *Public awareness* about the harmful effects of ageism and mental health stigma on the wellbeing of older adults can improve the perceptions of younger people about ageing and mental health [32]. Public awareness improves intergenerational interactions that buffer against social isolation and loneliness [41] and mental distress [28]. Affirmative interactions with other members of the community strengthen mutual respect, enhance social cohesion [46], and improve feelings of self-worth [35]. An extension of public awareness is *mental health literacy.* Improved mental health literacy across the life span can build the capacity of all community members to detect, manage, and seek treatment for mental health issues in self and others [47,48].

Meso (community programs and healthcare services): *Social empathy* can encourage positive intergenerational interactions and relationships [41]. A deep understanding of the life situations of others (including how these experiences are shaped by structural inequalities) [49] can produce positive attitudes about the contributions and value of older adults in younger workers and volunteers. Structural stigma can be mitigated when community and healthcare workers are provided with the knowledge and skills to recognize health stigma, understand how it is communicated and transmitted, and consider ways to disrupt stigma and discrimination in personal and professional situations [50]. Community and healthcare services/supports that create opportunities for *intergenerational connections* (including between those who do and do not experience mental health issues) can improve social empathy and foster mutual respect [29,46].

Micro (older adults): Efforts to build the *personal resilience* of older adults to cope with and counter the negative effects of stigma contribute to overall mental health and healthy ageing [51]. Resilience ‘thinking’ helps older adults cope with and adapt to stressful and adverse situations (e.g., social stigma, major life changes, bereavement). This in turn heightens their ability to protect against self-stigma [27], reducing the risk of depression and anxiety [28,52] and suicidality [38]. Resilience is indirectly associated with improved physical health, cognitive performance [53], and longevity [51]. An interconnection has also been shown between psychological resilience and improved healthcare transition experiences [54]. 

#### 4.3.4. Strategies for Action

Macro (community and healthcare systems): *Anti-stigma discrimination campaigns* can change the knowledge, attitudes, and behaviors of the general public regarding ageing and mental health [55]. Specific interventions include public education, mass media campaigns, and local community events that challenge stereotypes and highlight personal stories of stigma and discrimination [49,56]. Interventions can reduce levels of social and self-stigma and improve the ability of older adults to manage [48,57] and seek help for [47] mental health issues. *Mental health literacy,* when included as a component of these campaigns, can improve the knowledge, skills, and information of members of the public regarding the mental health of older adults [47]. 

Meso (community programs and healthcare services): *Sensitivity and awareness training programs* can reduce the stigmatizing attitudes and discriminating behaviors that older adults encounter in community and healthcare settings [50]. Specific interventions include in-service training sessions that involve formal education, role-playing, and personal story-telling [58]. Training programs help participants recognize stigma, understand how ageist and mental health stigmas are communicated and transmitted, and consider ways to disrupt stigma in personal, professional, and organizational practice [50]. Similarly, *intergenerational programming* is a way to create opportunities for the positive exchange of experiences between older adults and younger people to improve mutual respect and feelings of self-worth [59].

Micro (older adults): *Resilience-building initiatives* are applied therapeutic interventions that improve intrapersonal skills and help older adults remain psychologically ‘resilient’ in the face of adversity [60]. Specific interventions focus on building emotional awareness, strengthening coping capacity, fostering a sense of purpose, and creating supportive social networks [51]. There is a positive correlation between improved resilience and increased self-esteem, social connectedness, overall sense of wellness [60], and help-seeking [32] for older adults.

### 4.4. Domain 2: Age-Friendly Environments 

Environments can play an important role in either optimizing (or hindering) the overall functioning of older adults and can thus impact their ability to achieve healthy ageing [61]. Age-friendly environments (AFEs) are built and social environments that are accessible, equitable, inclusive, safe, and secure for older adults. The policies, services, and structures that shape AFEs intentionally support older adults to live safely, enjoy good health, and stay involved [62].

#### 4.4.1. Age-Friendly Environments and Mental Health

AFEs are an important catalyst for improving the mental health of older adults as a population [63]. AFEs are designed to ensure that older adults gain equitable access to social determinants of health (housing, food, security, employment, recreation and leisure, and health care) [35]. Many determinants of mental health harms including stigma and discrimination [29,35], social isolation [14,40], lack of physical activity [64,65], and income barriers [35,66] are thus mitigated by AFEs [67]. The features of AFEs that optimize mobility, physical activity, and meaningful social interactions are preventative of generalized anxiety [64], cognitive impairment [37], and social isolation and loneliness [68]. 

#### 4.4.2. Mental Health Harms

Macro (community and healthcare systems): Community and healthcare systems built on *infrastructures* (e.g., policies, standards, and regulations) that do not consider and respond to the requirements of older adults promote normative processes (e.g., ways of getting around, ways of working) that systematically hinder access to resources that support mental health and healthy ageing [61,63]. For instance, features in the built environment that commonly challenge older adults include a lack of inclusive transportation options [35], safe environments to engage in leisure and physical activity [34,65], and accessible public green spaces [65,66]. These missed opportunities can produce lower social capital (i.e., reciprocal social networks) [39,67] and increase psychological distress (Franke et al., 2020) and social isolation [41,68].

Meso (community programs and healthcare services): Community programs and healthcare services that do not consider the requirements of older adults carry forward the inequities fostered at macro levels [63]. For instance, when age-friendly language, strategies, and approaches are not incorporated into programs and services, older adults are less able to access supports that optimize their mental wellness [69,70,71]. They are also less likely to receive care that appropriately anticipates and responds to their mental health issues [72,73,74]. The *disadvantages* embedded in community and healthcare environments place older adults at greater risk of chronic illness, depleted social networks [75,76], and diminished quality of care [77,78]. Over time, older adults are less likely to seek out programs and services that could potentially mitigate mental health harms [79,80,81].

Micro (older adults): Environments planned without consideration of the requirements of older adults have a direct negative impact on their mental health and negatively impact how older adults are positioned socially, economically, and politically, thus, heightening experiences of *social exclusion and marginalization* [39,67]. For instance, environmental obstacles that impede meaningful social engagement and erode social networks heighten the risk of social [64] and socio-economic marginalization [35,66]. They also diminish opportunities to participate in civic life [40] (Zimmer and McDonough, 2022). Declines in the frequency, quality, and ability to influence social interactions have a negative impact on self-efficacy and self-esteem [34], increasing the risk of loneliness [82,83] depression [14,15], addictions [30], and suicidality [25,45]. 

#### 4.4.3. Optimizing Mental Health

Macro (community and healthcare systems): *Age-friendly community planning* can improve access to the social determinants of health for *all* older adults [63]. Age-friendly infrastructures promote environments that are accessible to older adults and that improve their mental health [82]. For example, situating natural open spaces so they are easily accessible and available to older adults contributes to population-level mental wellness outcomes [84]. Similarly, social environments that promote meaningful intergenerational engagement help older adults build social networks that lessen the risk of social isolation [64] and depression [14,15]. Healthcare systems built on policies, standards, and regulations that align with the requirements of older adults incentivize care processes that prioritize and address their mental health across the continuum of care [69].

Meso (community programs and healthcare services): The extent to which community programs and healthcare services are *designed to respond to the requirements of older adults* is correlated with improved access to quality services and supports. Engaging older adults in designing programs and services can improve not only accessibility but also the quality and relevance of available resources [85,86]. For instance, features of programs and services that enable older adults to enjoy frequent interactions with peers can improve opportunities for social participation [35], feelings of social attachment, life satisfaction, and psychosocial wellness [87]. The process of engaging in community planning efforts has been shown to improve self-confidence, self-esteem, sense of personal empowerment, and feelings of social contribution and worth on the part of older adults [85]. 

Micro (older adults)**:** When older adults are able to derive value from positive connections with others, they are able to harness *social capital*. Social capital is an asset that can help older adults access social, economic, and healthcare opportunities [68]. Social capital is a key factor that impacts mental health [88]. For example, older adults with high levels of social connectedness are more likely to report increased feelings of social cohesion, perceived adequacy of social support, and increased social motivation [89]. *Equitable access* to relevant and appropriate programs and services is critical for enabling adults to prevent and self-manage mental health issues [71]. Equitable access to natural open spaces for instance is a feature of AFEs that improves the emotional and physiological health of older adults [90].

#### 4.4.4. Strategies for Action 

Macro (community and healthcare systems): *Community engagement and consultation* with older adults can inform the geographical disbursement of valued resources [91]. Aligned with engaging older adults in planning efforts is the use of *universal design* (UD) principles [92]. UD mitigates the barriers that reduce independence and autonomy for older adults [93] and is positively associated with decreased experiences of ageism and discrimination [92]. UD can be used to plan *age-friendly healthcare environments* that improve the accessibility, quality, and appropriateness of care, and psychological wellness [94]. 

Meso (community programs and healthcare services): *Tailored programming* can help overcome barriers to access and participation by embedding unique features into the design of activities and programs (e.g., peer support, opportunities for socialization) that appeal to older adults [40]. Tailoring can improve the extent to which older adults attend and derive satisfaction from community programs [40,95] and build social capital [96]. Likewise, *tailored care delivery strategies* can produce improved therapeutic and quality-of-care outcomes [97,98].

Micro (older adults): *Companionship programs* can improve social participation, psychological wellness, quality of life, and overall functioning for older adults [99,100]. Interventions that nurture supportive interpersonal relationships improve feelings of belonging and wellness and improve social connectedness and psychological wellness [101]. Similarly, *health-generating neighborhood environments* that enable older adults to enjoy frequent interactions with neighbors can improve opportunities for social connectivity and maintain social networks [70,102,103]. 

### 4.5. Domain 3: Integrated Care

Integrated care is a collaborative approach to service delivery that aims to merge knowledge, resources, and practice across sectors (health, community and social services, public and private sector organizations and groups), layers (primary, secondary, tertiary, community), types (prevention, early intervention, outpatient, residential/inpatient), disciplines (nursing, medicine, physiotherapy, social work, pharmacy), and domains (physical, mental, social, environmental, and spiritual) of care [104]. The result is a comprehensive process that emphasizes a holistic, preventative, rehabilitative, and person-centered approach [105,106]. Integrated care is aligned with the goals of healthy ageing and is well suited for addressing the complex and intertwined health and social care requirements of older adults [11,107,108]. 

#### 4.5.1. Integrated Care and Mental Health

Integrated mental health care (IMHC) is a type of IC that aspires to merge mental health knowledge, resources, and practice across the continuum of care (i.e., sectors, layers, types, disciplines, and domains) to improve the prevention, detection, management, and treatment of mental health issues [109]. The integration of mental health care (i.e., services and support) across community and healthcare environments can improve mental health outcomes and care experiences for older adults as a population [2]. IMHC means that older adults have access to appropriate mental health care across settings (e.g., community-at-large, primary care, acute care, specialty care) to address their mental health requirements at any stage along their mental health journey (i.e., wellness, concerns, illness). IMHC has been shown to increase access to care, reduce mental health stigma, and improve help-seeking for mental health issues in older adults [110]. 

#### 4.5.2. Mental Health Harms

Macro (community and healthcare systems): Currently, there is *limited knowledge and expertise* across the continuum of care regarding the specific presentation and treatment of mental health issues in older adults [111]. Many providers in primary, LTC, home care, and acute care do not feel sufficiently equipped (e.g., knowledge, skills, resources) to deal (e.g., detect, assess, intervene) with mental health issues in older adults [112]. This affects the extent to which mental health issues can be prevented, detected, assessed, and treated in this population [113]. A service gap has also been noted regarding the availability of community support to help older adults (and families) locate and navigate their way to appropriate mental health services and support [114]. This is a situation that both impedes the timeliness in which mental health issues can be addressed and contributes to reduced help-seeking [114].

Meso (community programs and healthcare services): *Access barriers* that make it difficult to locate and receive appropriate mental health care across community and healthcare settings heighten the risk of mental health harms [107]. The lack of continuity in accessing mental health supports across community and healthcare environments delays referrals to mental health specialists [115], while delayed wait times to see mental health specialists elevate levels of stress and anxiety [116]. These are scenarios that delay treatment, worsen mental health symptoms [117], and contribute to premature placement in LTC facilities [118]. Moreover, a lack of seamless information-sharing between organizations and sectors creates critical gaps in mental health care that can worsen health harms [98]. An example is the overprescribing and inappropriate prescribing of medications for depression and anxiety that increase the risk of adverse drug events [118,119].

Micro (older adults): Older adults often receive *insufficient and inappropriate mental health care* [107]. If not addressed early, mental health concerns can manifest into more serious problems and disorders (i.e., illness) [119]. Moreover, prolonged psychological distress can exacerbate existing and precipitate new complications including chronic health conditions [119], social isolation, poor quality of life [120], and premature mortality [45]. There is building literature that suggests that a broader focus beyond disease-specific care is needed to fully address the mental health requirements of older adults. For instance, studies show that a strict focus on pharmacological treatment, without equal focus on therapeutic interventions (e.g., day-to-day coping, self-management), limits the overall effectiveness of mental health care [109]. 

#### 4.5.3. Optimizing Mental Health 

Macro (community and healthcare systems): Consistent and continuous access to quality mental health resources, approaches, and interventions across the continuum of care (i.e., within, between, and across health and social care professionals, organizations, and systems) can optimize the mental health of older adults as a population [107]. Integrated health and social systems (IHSSs) (and connected infrastructures) can promote equitable access to appropriate, consistent, and continuous mental health care. IHSSs are networks that support joint planning, funding, and inter-operations between and across diverse community- and healthcare-based organizations [121]. IHSSs can support inter-organizational and inter-sectoral collaboration, which increases the accessibility and continuity of mental health care across the continuum of care [122] and improves the prevention, detection, management, and treatment of mental health issues in the older adult population [110].

Meso (community programs and healthcare services): It is increasingly recognized that health promotion and prevention strategies broaden the scope of mental health services and supports [122] (ref). *Mental health prevention and promotion* in and across community and healthcare settings are increasingly recognized as a way to support optimal physical, emotional, cognitive, and social functioning and enable the day-to-day management of mental health concerns [123]. Improvements in functioning and self-management are shown to have interrelated effects on mental health and wellbeing [124]. The adoption of a mental health prevention and promotion approach across the continuum of care is increasingly seen as a way to reduce service delivery redundancies, close care gaps, and improve the navigation experiences of older adults [125].

Micro (older adults): An *older-person-centered approach* is enacted when programs and services take into account the preferences, capacities, and overall wellbeing of older adults [110]. For instance, when community programs and healthcare services are delivered in ways that support older adults to maintain their independence, autonomy, and choice, they improve mental health and wellbeing [17]. Similarly, healthcare services that include prevention and self-management of components help preserve mental wellness and support the everyday management of mental health issues [126]. Applied positive psychiatry initiatives have shown promise to improve the mental health and wellbeing of individual older adults [5].

#### 4.5.4. Strategies for Action

Macro (community and healthcare systems): *Collaborative community health partnership initiatives* can bring multiple stakeholders and influencers together across organizations and sectors to leverage resources and build bridges that boost mental health equity [127]. These initiatives involve bringing community and healthcare stakeholders together to generate solutions, leverage resources, and take collective action to fill local community/health service gaps. Community health partnerships can help develop age-friendly communities that support mental wellness and provide easy access to support that helps older adults cope with mental health concerns and address mental illnesses [128]. 

Meso (community programs and healthcare services): *Models of integrated mental health care* enable service providers across diverse care settings (e.g., primary care, acute care, home care, LTC, specialty geriatric mental health care) to co-plan, co-coordinate, and co-deliver mental health services and supports [121]. Specific strategies include inter-organizational circles of care teams, lunch and learn sessions, and cross-training initiatives [129]. Technology-enhanced mental health services (including telepsychiatry, mHealth applications, and shared electronic health records) are a key component of IMHC that help broaden the reach of mental health knowledge, resources, and practice across the continuum of care [130,131]. Applied positive psychiatry interventions, when accessible on a public health scale, are associated with improvements in mental health wellbeing and promotion outcomes for older adults as a population [5].

Micro (older adults): Difficulties locating appropriate mental health supports can increase psychological distress (Funk et al., 2019) and decrease help-seeking [48]. *Community-based navigation hubs* can facilitate help-seeking, improve timely referrals, and fill gaps in mental health services [132]. Navigation hubs are ideally centrally located in community settings. They aim to help older adults navigate and access appropriate community and healthcare programs/services to support mental (and other) health issues. For older adults, reducing navigation difficulties can increase levels of help-seeking and improve the efficiency and quality of mental health care [57]. 

### 4.6. Domain 4: Long-Term Care

Older adults who experience functional limitations often require longer-term health (i.e., prevention, treatment, maintenance of a health issue or condition) and social (i.e., providing assistance with activities of daily living, maintaining independence) support [133]. The continuum of long-term care (LTC) (also referred to in many jurisdictions as continuing care) supports encompasses in-home services (e.g., in-home nursing care), community-based ageing-in-place options (e.g., alternative models of independent living designed to support ageing-in-place), and LTC facilities (i.e., residential care homes) [134].

#### 4.6.1. LTC and Mental Health

The extent and quality of LTC can have a direct impact on the mental health of older adults [135]. Clear links have been identified between unmet LTC needs and increased psychosocial distress, poor overall health, increased use of emergency health services, and premature admission to LTC facilities [133]. Moreover, a high prevalence of mental health issues and conditions has been found among older adults who receive in-home LTC support and those who reside in LTC facilities [136].

#### 4.6.2. Mental Health Harms 

Macro (community and healthcare systems): Studies show that older adults who receive LTC support are at a heightened risk of experiencing poor quality of life and elevated mental health harms [137]. However, there is *insufficient mental health support* available for these older adults. Mental health support in LTC facilities is largely provided by mental health specialists on a consultation basis. This means that mental health issues must first be recognized and reported by LTC staff who may lack training in detecting mental health issues [109]. Similarly, the unmet mental needs of older adults who receive LTC in the home environment precipitate avoidable emergency room visits and premature placement in LTC facilities [134]. The lack of desirable and affordable alternate living options for older persons who require more intensive support but who wish to remain living in the community can intensify emotional distress and apathy [138,139]. 

Meso (community programs and healthcare services): LTC supports emphasize medical and nursing support and place less *emphasis on promoting independence and autonomy* (e.g., mobility, emotional coping, self-management skills) [140]. This can produce declines in overall functioning [11] that decrease motivation, increase psychological distress, and contribute to poor quality of life [28,60]. Likewise, many aspects of LTC facilities (e.g., congregate living, limited opportunities for physical activity, reduced time with family and friends, limited autonomy) have been shown to curtail independence and autonomy for residents [141]. The de-emphasis on physical and social functioning in LTC facilities is correlated with increases in emotional distress [142] including clinical depression, generalized anxiety, and impaired cognition [14].

Micro (older adults): Studies show that most older adults prefer to remain living in the community [143] and can experience heightened psychological distress in anticipation of moving to an LTC facility [144]. LTC contexts that contribute to a *loss of functionality and self-determination* decrease physical and social participation [28] and elevate psychological distress [145]. The health of older adult caregivers can also be negatively impacted when their health and care needs are not considered within LTC contexts [146]. Long-term caregiving is shown to incite mental health harms including depression and anxiety [115] and associated declines in cognitive performance [147].

#### 4.6.3. Optimizing Mental Health

Macro (community and healthcare systems): Recently, there have been efforts to design *LTC environments that promote independence and autonomy* [148,149]. These models have the potential to close care gaps and alleviate mental health harms [15]. Studies indicate that alternative forms of adapted living that incorporate features attuned to the requirements of older adults (e.g., affordability, self-governance, meaningful social participation, and engagement) can reduce social isolation and loneliness [70] and prevent or reduce depression and anxiety [39]. Similarly, when LTC facilities are designed with home-like features (e.g., small-scale, home-like furniture, brightly colored spaces, décor, plants), they can have a positive impact on the psychological wellness of residents [150,151]. 

Meso (community programs and healthcare services): There is evidence that the *integration of mental health services/supports* into LTC contexts can improve mental health and care outcomes [135,152,153]. Providing routine screening for mental health issues to individuals who receive LTC support (in-home care and LTC facilities) can prevent or delay the onset and exacerbation of mental health issues [132]. Likewise, incorporating mental health support to family caregivers can improve their overall mental wellness and quality of life [154]. 

Micro (older adults): Research shows that most older adults prefer a holistic model of LTC that incorporates support for overall functioning, social participation [140], and engagement in meaningful life activities [154]. LTC support that focuses on functionality and self-management in home- and facility-based care can improve mental health and wellbeing outcomes [118], for instance, support that aims to improve movement, mobility [84], and performing activities of daily living [155] and reduce experiences of depression and anxiety [140,156].

#### 4.6.4. Strategies for Action

Macro (community and healthcare systems): *Alternative LTC and ageing-in-place model* designs that support independence and autonomy also promote mental wellbeing [137,157]. For instance, naturally occurring retirement communities (NORCs) are shown to improve mental and physical health, motivation, and life satisfaction [158]. When supportive home care and community support are embedded into these environments, older adults are able to remain living in the community as long as possible and avoid or delay the transition to an LTC facility. Similarly, innovative architecture (signage, color, space, light, and views of nature) in LTC facilities can better enable social interactions, physical activity, and time spent in nature [28,140,159], contributing to mental wellness and overall life satisfaction [160]. 

Meso (community programs and healthcare services): *Integrating mental health care into LTC contexts* can reduce the frequency and severity of psychiatric symptoms (e.g., agitation, aggression, depression, anxiety, disinhibition) [136] and improve the overall quality of life [160]. There are different ways to integrate mental health knowledge, resources, and practice into LTC contexts. Some models heighten access to visiting or on-call psychiatrists, and/or nurse specialists, to assess, plan, and implement appropriate mental health interventions that are then carried out by LTC staff [136]. Others involve interdisciplinary mental health consultation through in-person visits or via telepsychiatry [140]. Educational training sessions provided to LTC staff can strengthen core competencies in identifying and responding to mental health symptoms and issues [161].

Micro (older adults): Holistic care approaches that *emphasize functionality and self-management* can help older adults remain in their homes longer, delay admission to LTC facilities, and improve psychological distress and overall wellness [162,163,164]. For instance, simple exercises prescribed by a physiotherapist and facilitated by home care staff can improve functional performance, personal motivation, and perceptions of wellbeing [165,166]. Similarly, incorporating physical and social activity programming into LTC facilities can positively influence overall mental health [167,168]. Self-management interventions incorporated into home care contexts optimize the management of mental health issues in addition to other health concerns [169]. 

## 5. Discussion

The Framework described in this paper structures relevant concepts, elements, and strategies into an analytical tool intended to guide transformative change across community and healthcare domains to optimize the mental health of older adults in support of healthy ageing. The Framework contextualizes the four areas (or domains) of the *Decade* in terms of older adult mental health. Mental health is positioned along a continuum that encapsulates mental wellness, mental health concerns, and mental illness. Moreover, mental health is considered an interlinking mechanism that determines (and is determined by) physical, social, environmental, and spiritual health. The Framework operationalizes each domain by way of three components: barriers (mental health harms), facilitators (optimizing mental health), and strategies (for action). Each component is subsequently organized into macro, meso, and micro levels of influence. 

The concepts and components of the Framework were informed by an extensive review and analysis of the peer-reviewed and grey literature. The research articles included in this study were not assessed and ranked in terms of methodological strength, nor were concepts and strategies weighted in terms of level of importance or causal directionality. 

Several base assumptions that underlie the Framework are unique from those of other F frameworks in the literature that focus on the mental health of older adults in the context of healthy ageing [2,4,112,169]. The current Framework presumes that (a) at any given time, *all* older adults experience mental health issues in some form; (b) the mental health experiences of individuals are not fixed and can fluctuate (back and forth) along a continuum; (c) mental health is impacted by combinations of environmental, circumstantial, and physiological stimuli; (d) barriers, facilitators, and strategies for action exist in and across community and healthcare domains; and (e) mental health is a fundamental component of and catalyst for healthy ageing. The Framework also highlights the barriers and opportunities at play within, between, and across macro (social and health systems), meso (community programs and healthcare services), and micro (older adults) levels of influence. 

The concept of mental health as a foundational component of healthy ageing is important. It identifies the role of mental health as a critical enabler (or disabler) of healthy ageing. The view of mental health as being similar to that of physical health (i.e., a dynamic, fluid, and ever-present condition) is equally important in that it makes the need to support the mental health of *all* older adults visible. In the current Framework, mental health is, thus, re-positioned as a population health issue rather than an issue reserved only for individuals who live with or are at risk of mental illness. This transformative lens underscores the need to provide *all* older adults with the tools and resources to prevent, manage, and treat mental health concerns and illnesses as a key component of a broader healthy ageing strategy. The Framework highlights the intersectional barriers and facilitators that operate in and across community and healthcare domains. The responsibility for older adult mental health is, thus, dispersed across the multiple and diverse community and healthcare environments that shape everyday lives. It is this dispersing of responsibility beyond the healthcare domain that makes real transformation in the mental health experiences of older adults possible. Finally, the identification of strategies that can be applied in and across domains at macro, meso, and micro levels of influence pinpoints a strategic pathway to action new realities that optimize older adult mental health. 

The current Framework is intended to provide a pluralistic and connected approach to (re) conceptualize, (re) organize, and (re) structure efforts to optimize the mental health and healthy ageing trajectories of older adults. It offers an alternative way of situating mental health that resists dividing mental health experiences into distinct compartments (e.g., wellness vs. concerns vs. illness) and re-distributes responsibility for its optimization across community and healthcare domains. The Framework is applicable to decision leaders and practitioners who operate in policy, practice, education and training, research, and knowledge mobilization arenas in both community and healthcare domains. It is hoped that the thinking and strategies contained in the Framework will encourage collaborative efforts (in and across arenas and domains) that can lay the foundation for an integrated community/healthcare infrastructure that better enables older adults to attain and preserve good mental health as well as access appropriate resources (across all environments) to treat mental health concerns and illnesses.

## 6. Conclusions

To improve population-level healthy ageing, the mental health of *all* older adults must be considered a priority in policy, practice, education and training, research, and knowledge mobilization arenas in and across community and healthcare domains. We aimed to develop a pluralistic Framework to inform transformative change across community and healthcare domains to optimize the mental health of older adults in support of healthy ageing. The resulting Framework is applicable to countries and jurisdictions that wish to apply a pluralistic and connected approach to transform the mental health and healthy ageing outcomes of older adults as a population. It can be used by decision leaders and practitioners operating in policy, practice, education and training, research, and knowledge mobilization arenas in and across community and healthcare domains to set priorities, build evidence, and scale up promising practices nationally and sub-nationally. 

## Data Availability

Further inquiries can be directed to the corresponding author.
